# Validation of the Sexual Inhibition/Sexual Excitation Scales (SIS/SES) in Italy: Assessing Gender and Age Differences of Sexual Functioning

**DOI:** 10.1007/s10508-021-01972-3

**Published:** 2021-08-06

**Authors:** Marta Panzeri, Harold Dadomo, Lucia Ronconi, Lilybeth Fontanesi

**Affiliations:** 1 Department of Developmental Psychology and Socialisation, Università Degli Studi di Padova, via Venezia 8, 35131 Padua, Italy; 2 Unity of Neuroscience, Department of Medicine and Surgery, University of Parma, Parma, Italy; 3 FISPPA, Università degli studi di Padova, Padua, Italy; 4 Department of Psychological, Humanistic and Territorial Sciences, University of Chieti, Chieti, Italy

**Keywords:** SIS/SES, Sexual excitation, Sexual inhibition, Gender differences, Age differences

## Abstract

The Sexual Inhibition Scales and Sexual Excitation Scales (Janssen et al., 2002a), based on the dual control model by Bancroft and Janssen (2000), are part of a 45-item self-report questionnaire evaluating individual tendencies to sexual inhibition or excitation according to three factors: two inhibition factors, SIS1, threat of performance failure, and SIS2, threat of performance consequences, and one excitation factor, SES. In this paper, we aimed to validate and explore psychometric properties of the SIS/SES in a sample of 2260 Italian men and women aged 18 to 75 years. Confirmatory factor analyses showed that the three-factor structure proposed in the original version of the scales fit with our sample. Moreover, our data confirmed the results of the original validation sample: Women scored higher on the SIS and lower on the SES than men did, but no significant differences appeared in the factor scores by age group, except for a gender × age interaction, where younger women had higher SIS2 scores. The SIS/SES appeared to be an effective, appropriate cross-cultural measurement of human sexuality in Italian samples, also shedding light on sexual arousal differences in women and men in our country. We also discuss clinical and therapeutic aspects.

## Introduction

Researchers have studied sexual arousal as an important stage in sexual response cycles for decades, ever since Masters and Johnson (1966) described it. Recently, sexual arousal has been conceptualized as the outcome of an adaptive process involving psychological, physiological, and behavioral aspects, as synthesized in the dual-control model (DCM) proposed by (Bancroft & Janssen 2000; Bancroft et al., 2009; Janssen & Bancroft, 2007). The DCM focuses on the assumption that sexual arousal is the product of a complex integration between inhibitory and excitatory mechanisms of the central nervous system (Bancroft, 1999; Bancroft & Janssen, 2000; Janssen & Bancroft, 2007). People vary in their propensity for sexual excitation (SE) and sexual inhibition (SI), and these two aspects have been determined to be independent of one another (Janssen et al., 2002a, 2002b). Individuals with a high propensity for excitation and/or a low inclination for inhibition are more likely to engage in high-risk or problematic sexual behavior, such as unwanted pregnancies and sexually transmitted diseases (Macapagal et al., 2011). In contrast, people with a low propensity for SE and/or a high propensity for SI are expected to face problems with sexual response impairment, of which sexual dysfunctions are an example (Bancroft, 2009). Sexual functioning complications and sexual risk-taking behaviors are health-related issues of significant personal, relational, and social concern, and the DCM can create a conceptual framework for the way individual differences contribute to these situations (Unterhorst et al., 2020).

Much research on the DCM used the Sexual Inhibition and Sexual Excitation Scales (SIS/SES; Janssen et al., 2000a) to highlight how the DCM can regulate sexual behavior and to study individual differences in sexual response (i.e., propensity for SE and SI) in various cultures and human populations (Bancroft & Vukadinovic, 2004; Bancroft et al., 2003, 2004, 2005a, 2005b; Janssen et al., 2002a, 2002b).

Moreover, the SIS/SES recently has been used successfully to study the relationship between excitatory and inhibitory systems, hypersexuality (Rettenberger et al., 2016; Walton et al., 2017), and sexual dysfunctions in males and females (Quinta Gomes et al., 2018; Tavares et al., 2018; Turner et al., 2019). The SIS/SES also appears to be a valuable tool for measuring sexual arousal and inhibition (Bancroft et al., 2003, 2005a, 2005b), male sexual dysfunction (Bancroft et al., 2005a, 2005b), male sexual compulsivity (Bancroft & Vukadinovic, 2004), risky sexual behaviors among homosexual and heterosexual men (Bancroft et al., 2004), and sexual arousal, highlighting the differences between male and female sexual systems (Carpenter et al., 2008).

The SIS/SES consists of three factors: one that measures sexual arousal (SES) and two that measure sexual inhibition. The first inhibition factor (SIS1) is related to the level of inhibition and is associated with the fear of failure in sexual performance (Bancroft & Janssen, 2000). According to Bancroft and Janssen, the second inhibition factor (SIS2) is related to the fear of external threats. Of these two inhibitory systems, SIS1 has been suggested to represent vulnerability to sexual dysfunction (e.g., Bancroft et al., 2004). SIS2, on the other hand, is more relevant to vulnerability to risky behavior. Therefore, low SIS2 scores imply sexual arousal that is not inhibited by evidence of risk, compromising risk management. Sexually transmitted diseases, unwanted pregnancy, and legal consequences are therefore related to this factor (Bancroft et al., 2004).

The SIS/SES in either the original or the short form has been validated in many countries and translated into different languages, such as German (Rettenberger & Briken, 2013), Spanish (Moyano & Sierra, 2014), Dutch (van Lankveld et al., 2015), and many South Asian languages, such as Hindi, Urdu, Panjabi, Tamil, and Sinhalese (Malavige et al., 2013). The 14-item short version was recently validated in German (Rettenberger et al., 2019; Velten et al., 2018), and the 45-item version was validated in Finnish (Varjonen et al., 2007), Portuguese (Quinta Gomes et al., 2018), and Spanish (Granados et al., 2018). The Sexual Excitation/Sexual Inhibition Inventory (SESII) for Women (a different instrument developed from focus group analysis, assessing only female systems; Graham et al., 2004) has been adapted in Dutch (Bloemendaal & Laan, 2009), German (Velten et al., 2018), Polish, Portuguese (Neves et al., 2016), and Spanish (Granados et al., 2017), and the SESII for Men has been adapted in German (Velten et al., 2018) and Portuguese (Neves et al., 2016).

The original validation showed good psychometric properties: even if the model with better fit indexes was the 10 level model, Janssen et al. (2002a) preferred the 3-in-10 model, whose fit indexes were only slightly different from the 10 level model, for its better fit in research and clinical practice (Janssen et al., 2002a). Factorial invariance for gender was also confirmed (Carpenter et al., 2008). The German validations (Rettenberger et al., 2019), which regarded only the SIS/SES short form (Carpenter et al., 2011), showed good psychometric properties and confirmed the original results and factorial invariance for gender. The Finnish validation (Varjonen et al., 2007) eliminated nine items for various reasons, confirming the structure of the three superordinate factors (SES, SIS1 and SIS2) but finding 25 lower-order factors instead of the 10 original ones found by the authors (Janssen & Bancroft, 2007). The Spanish validation (Granados et al., 2018) eliminated 11 items for which the factor loadings were too low and added a third inhibition factor, related to the threat of performance consequences, that had insufficient internal consistency. The authors claimed that this third inhibition factor, even without sufficient reliability, was closely linked to the DCM assumption that SI is an adaptive phenomenon protecting women from negative consequences such as an unwanted pregnancy or pain due to intercourse. The Portuguese validation (Quinta Gomes et al., 2018) showed good psychometric indices and confirmed the original structure. To measure possible sexual dysfunctions, the authors administered two other tests: the International Index of Erectile Function to men (IIEF; Rosen et al., 1997) and the Female Sexual Function Index to women (FSFI; Rosen et al., 2000). Multiple regression analyses using the SIS/SES, and age as independent variables and the IIEF or FSFI as dependent variables have been carried out. Results showed that for men, SIS1 and age were negative predictors of sexual desire, erectile function, and orgasms, and SES was a positive predictor of sexual desire and erectile function; for women, SES was a positive predictor of sexual desire, arousal, lubrication, and orgasm, and age was a negative predictor of sexual desire. The authors noted that no study so far has investigated relationship aspects or emotional intimacy, variables known to be related to SE and SI (Basson, 2000, 2002; Byers, 2005).

These results showed that gender-related differences were found in relation to SIS/SES, and SESII subscales in every sample of every country, but regarding sexual orientation differences, the literature is quite lacking in significant results. Regarding homosexual women, for example, sexual orientation did not influence women’s sexual function, and the relation between SIS/SES and sexual function was the same as for heterosexual women (Carpenter et al., 2008; Jozkowski et al., 2016). Similar differences in the SIS/SES and the expression of sexual function, sexual arousal, and sexual behaviors have been found to be related not to sexual orientation in males but to other personal and developmental factors (Bancroft et al., 2005a, 2005b; Janssen et al., 2009).

To provide a reliable measure for the Italian population, we performed the Italian SIS/SES validation in this study. We expected to replicate the results obtained in the United States and Portugal, confirming the effectiveness of the SIS/SES. Our objective was to validate the SIS/SES in Italy following the original work by Carpenter et al. (2008) and highlight its psychometric proprieties in a sample of women and men, independently of their sexual orientation or gender identity.

Because no previous study has investigated different age groups, we also aimed to confirm structural invariance related to age in men and women. Growing attention has been paid to elderly sexuality (Štulhofer et al., 2019; Syme et al., 2018) and sexual changes related to different ages in men and women (Janssen et al., 2008; Pappalardo & Panzeri, 2015; Pinxten & Lievens, 2014). We also aimed to analyze the convergent and discriminant validities of the SIS/SES scales. Because no previous study has investigated SIS/SES regarding couples’ relationships, although Quinta Gomes et al. (2018) suggested its relevance, we decided to introduce this novelty in our study.

We expected the original factor structure would hold in Italian and the factor invariance between men and women would be supported by confirmatory factorial analyses (CFAs), in line with American (Carpenter et al., 2008) and European research on the subject (Pinxten & Lievens, 2014; Quinta Gomes et al., 2018; Velten et al., 2018). We also expected to find evidence of factor invariance between different age ranges.

Additionally, we expected to find gender-related differences in the SIS/SES (Carpenter et al., 2008; Pinxten & Lievens, 2014; Velten et al., 2018). Males should display higher scores in the SES and females in the SIS. This variability has been proposed by evolutionary psychology theories on innate gender differences in sexual behavior (Bjorklund & Kipp, 1996; Fontanesi & Renaud, 2014; Shackelford & Goetz, 2007; Symons, 1979). Because age negatively impacts sexuality (Ferrucci et al., 2016; Janssen et al., 2002a), we expected to find age differences in the SIS/SES. Younger participants should display higher scores in the SES and older ones in the SIS due to the decrease in testosterone in men and the adverse effects of menopause in women (Bancroft, 2009).

Finally, we expected that the SIS/SES would be a good predictor of sexual dysfunction in women and men, because people with high levels of SES and low levels of SIS are prone to developing sexual dysfunctions, as the DCM postulated (Janssen et al., 2002a). Because clinically assessing sexual dysfunctions was not possible, we chose to use the IIEF and FSFI similar to previous studies (Bancroft et al., 2009; Quinta Gomes et al., 2018; Sanders et al., 2008) to be able to compare our results with previous ones.

## Method

### Participants and Procedure

We informed all participants about the purpose of the study and received written informed consent. The complete data set of participants who completed the SIS/SES consisted of 3337 women and men. The ones whose age was known numbered 3275. We recruited two samples to obtain data on different age groups (one each of younger and older participants). Sample 1 consisted of 2032 undergraduate students, of whom 61 did not write their age on the preliminary personal and social particulars form, and three completed SIS/SESs were not valid according to the authors’ Script Scoring (which reversed some scores and eliminated subjects missing more than 10 answers total, more than 5 SES, more than 4 SIS1, and more than 3 SIS2), leaving us with 1968 subjects. Sample 2 consisted of 1317 participants: one participant did not write their age, and 12 completed SIS/SESs were not valid, according to the authors’ Script Scoring, leaving us with 1304 subjects.

### Sample 1

The first sample consisted of 1968 undergraduate students (891 males, 1077 females), less than 35 years of age, from Italian universities located in different parts of Northern, Southern, and Central Italy. For this study, we used a convenience sample of university students. Questionnaires were administered in class during a regular class period by five trained graduate students supervised by an expert researcher after an explanation of the study’s aims (validation of the instrument). The participants had to read and sign a written consent form. At the end of the session, students had to put the questionnaires in a closed box. The mean age for men was 23.92 (*SD* = 3.54); the mean age for women was 22.06 (*SD* = 2.77). Both had an age range of 18 to 35 years. This sample was tasked with completing the SIS/SES.

### Sample 2

The second sample consisted of 1292 participants extracted from the general population. Questionnaires were administered by trained graduate students supervised by an expert researcher. The participants were contacted personally. The general aim of the study was explained to them verbally and reported in the written consent form, and their participation in the research was subjected to the signing of this document. To guarantee participants' identity and privacy were concealed, the tests were collected in a sealed envelope. Snowball and word-of-mouth sampling methods were used to recruit participants. Of those participants, 611 were male, and 681 were female. The mean age for men was 34.51 (*SD* = 10.85, range 18–75); the mean age for women was 33.22 (*SD* = 10.52, range 18–70). Dividing Sample 2 into two age groups (18–33 and 34–75 for men; 18–33 and 34–70 for women), we obtained mean ages of 26 (*n* = 305) and 43 (*n* = 306) for men, respectively, and we obtained mean ages of 26 (*n* = 385) and 43 (*n* = 296) for women. We found no significant differences regarding marital status within the sample.

A first subsample (Sample 2a, *n* = 70) was required to complete only the SIS/SES. Along with the SIS/SES, all of the other participants were required to complete the Brief Index of Sexual Functioning for Women (BISF-W; Taylor et al., 1994; Italian validation Panzeri et al., 2009) or the Brief Index of Sexual Functioning for Men (BISF-M; Panzeri & Raoli, 2010). Second and third subsamples (Samples 2b) were required to complete the Quality of Marriage Index (QMI; Norton, 1983) and FSFI (Rosen et al., 2000; Sample 2bf), and the QMI and IIEF (Rosen et al., 1997; Sample 2bm), respectively. These two subsamples consisted of 192 and 194 participants, respectively. The inclusion criterion was that participants had been in an exclusive relationship for at least 2 years.

Table 1 shows the sexual orientation distribution of our samples. This research accounts for a population-based sampling, and the number of homosexuals and bisexuals reflects the physiological percentage of not-heterosexual people in our country (ISTAT, 2011) even if the percentage is higher in the sample of university students. Due to the small number of people of homosexual orientation in our sample, performing an analysis of sexual orientation differences in the samples was not possible.

**Table 1:** Descriptive data

		Sample 1 (Students)			Sample 2 (General Population)		
		Men [N (%)]	Women [N (%)]	Total [N (%)]	Men [N (%)]	Women [N (%)]	Total [N (%)]
*Marital status* (*N1* = 1968*; N*2 = 1284)	Single	830 (93.2)	1008 (93.7)	1838 (93.7)	342 (56)	362 (53.4)	704 (54.8)
	Cohabiting/married	49 (5.5)	55 (5.1)	104 (5.3)	229 (37.8)	268 (39.5)	497 (38.7)
	Separated/divorced	4 (0.5)	12 (1.1)	16 (0,8)	32 (5.3)	41 (6.0)	73 (5.7)
	Widowed	2 (0.3)	1 (0.1)	3 (0,2)	3 (0.5)	7 (1.0)	10 (0.8)
*Relationship*	Monogamous	474 (53.6)	697 (67.4)	1171 (61.1)	421 (69.7)	526 (77.8)	947 (74.0)
(*N*1 = 1917; *N2* = 1280)	Nonexclusive	180(20.4)	101 (9.8)	281 (14.7)	77 (12.7)	58 (8.6)	135 (10.5)
	No current relationship	230 (26.0)	235 (24.3)	465 (24.3)	106 (17.5)	92 (13.6)	198 (15.5)
*Scholarity*	Middle school	–	–	-	90 (14.8)	58 (8.5)	148 (11.5)
(*N1* = 1972; *N2* = 1289)	High school diploma	891 (100.0)	1077 (100.0)	1968 (100.0)	254 (57.9)	423 (61.8)	677 (52.5)
	Undergraduate degree	–	–	–	138 (22.6)	190 (27.9)	328 (25.4)
	Graduate degree	–	–	–	27 (4.4)	11 (1.6)	38 (2.9)
*Orientation* (N1 = 1648; N2 = 1159)	Hetero/straight	643 (93.2)	903 (94.3)	1546 (93.8)	499 (92.2)	588 (95.1)	1087 (93.8)
	Homosexual/gay	15 (2.2)	6 (0.6)	21 (1.3)	21 (3.9)	13 (2.1)	34 (2.9)
	Bisexual	5 (0.7)	11 (1.1)	16 (1.0)	8 (1.5)	2 (0.3)	10 (0.9)
	Uncertain	27 (3.9)	38 (4.0)	65 (3.9)	13 (2.4)	15 (2.4)	28 (2.4)
*Sexual activity* (*N1* = 1917; *N2* = 1284)	Ever had opposite-sex partner?	861 (98.9)	1043 (99.7)	1904 (99.3)	590 (97.4)	670 (98.8)	1260 (98.1)
	Ever had same-sex partner?	31 (3,6)	33 (3.2)	64 (3.3)	38 (6.3)	28 (4.1)	66 (5.1)

The questionnaire responses were double entered, with one person dictating and checking the correctness of the output and another entering the data. The protocol for this study was reviewed and approved by the Institutional Review Board of the University of Cassino and Southern Lazio.

### Measures

#### Sexual Inhibition and Sexual Excitation Scales

The 45 items of the SIS/SES consist primarily of “if... then” statements (e.g., “If I am on my own watching a sex scene in a film, then I quickly become sexually aroused.”). Each item was rated on a four-point Likert scale (1 = *I strongly agree*, 2 = *I agree*, 3 = *I disagree*, and 4 = *I strongly disagree*). The three factors had a close to normal score distribution. Each scale’s middle-range showed a normative response, and the extremes showed a dysfunctional response.

The SIS/SES scales form an instrument that measures individual SE and SI. SE is measured by one factor (SES) that describes sexual arousal related to social interactions, fantasies, visual stimuli, or even nonsexual situations (e.g., bathing or sunbathing). SI is measured by two factors: SIS1, inhibition due to threat of performance failure, and SIS2, inhibition due to threat of performance consequences (Janssen et al., 2002a). SIS1 focuses on SI related to a threat of performance failure (e.g., arousal difficulties, loss of arousal, concern for the partner’s pleasure, etc.). SIS2 focuses on SI due to the threat of consequences related to sex (e.g., the risk of being caught, sexually transmitted diseases, pain, unwanted pregnancies, etc.; Janssen et al., 2002a). A study on a sample of 459 undergraduate men and a sample of 313 men recruited from faculty and staff confirmed the three-factor structure (Janssen et al., 2002a). The three-factor structure was also confirmed in a sample of 1,067 undergraduate women and 978 undergraduate men (Carpenter et al., 2008). This research also demonstrated the factorial invariance for gender in the SIS/SES. Males had higher scores on the SES than women but scored lower on SIS1 and SIS2. In 2011, a 14-item short version of the SIS/SES was developed to be used in situations requiring quick administration (Carpenter et al., 2011).

Intercorrelations have indicated that the excitation factor (SES) and the two inhibition factors (SIS1 and SIS2) were relatively independent (Carpenter et al., 2008; Janssen et al., 2002a, 2002b). SIS1 and SIS2 showed a low correlation in men (*r* = 0.26) and women (*r* = 0.19); this suggests that the scales measure separate constructs. The SIS/SES scores also showed acceptable test–retest reliability (*r* = 0.67 and *r* = 0.76 for the two samples) and good convergent and discriminant validities (Janssen et al., 2002a, 2002b). The Cronbach’s alpha results presented high internal consistency, with scores of 0.88, 0.80, and 0.71 for men and 0.87, 0.76, and 0.70 for women for the three factors, respectively (Carpenter et al., 2008). Similar results were found in the Finnish (van Lankveld et al., 2015) and the Portuguese (Quinta Gomes et al., 2018) versions of the SIS/SES and in the German validation (Rettenberger et al., 2019; Velten et al., 2018) of the short form of the instrument, but in the Spanish version (Granados et al., 2018), the results were slightly different due to the 11 items being eliminated and one inhibition factor being added.

The results of these studies proved the SIS/SES to be a reliable instrument for assessing sexual arousal, highlighting the differences between male and female sexual systems. The SIS/SES was translated into Italian using forward and backward translation done independently by two researchers with a high level of English proficiency and an in-depth comprehension of both cultures. Several sex researchers evaluated the cross-cultural equivalence of the Italian and English versions of the SIS/SES, focusing on individuating the most appropriate words and phrases. A final version of the instrument was tested for understandability and appropriateness in a small group of graduate students.

#### Brief Index of Sexual Functioning for Men and Brief Index of Sexual Functioning for Women

The BISF-W (Taylor et al., 1994; Italian validation by Panzeri et al., 2009) is a 22-item self-report questionnaire designed to investigate sexual function. Most items are arranged in a Likert-type format to rate the frequency of the occurrence of sexual desire, arousal, or satisfaction. Four major factors have been identified: couple sexuality (Factor 1), autoeroticism (Factor 2), dissatisfaction, (Factor 3), and anal sexuality (Factor 4). A version of the BISF has been developed for men, to study sexual function in couples (Panzeri & Raoli, 2010). The Italian validation of the BISF-W presented good internal consistency, with Cronbach’s alpha results of 0.95 for Factor 1, 0.85 for Factor 2, 0.73 for Factor 3, and 0.80 for Factor 4 (Panzeri et al., 2009). The BISF-M also presented good internal consistency: Cronbach’s alphas were 0.94 for Factor 1, 0.89 for Factor 2, 0.75 for Factor 3, and 0.83 for Factor 4 (Panzeri & Raoli, 2010). A quantitative scoring algorithm was developed to facilitate the use of the BISF-W in clinical trials (Mazer et al., 2000). This scoring procedure provides an overall composite score for sexual function, as well as seven dimension scores: relationship satisfaction, thoughts/desire, arousal, frequency of sexual activity, receptivity/initiation, pleasure/orgasm, and problems affecting sexual function.

#### International Index of Erectile Function

The IIEF (Rosen et al., 1997) is a 15-item self-report multidimensional scale used to assess erectile dysfunction and related factors. It investigates five interrelated domains: erectile function, orgasmic function, sexual desire, intercourse satisfaction, and overall satisfaction. All subscale scores are summed up in a total score. All items are scored on a 0–5 range. The test has strong internal consistency, with Cronbach’s alpha values of 0.73 and higher for the five main domains and 0.91 and higher for the total scale. The Italian linguistic validated version was used (Rosen et al., 1997), unless there was no Italian validation of the IIEF but only an Italian adaptation widely used all over the country, as can be found in the literature (e.g., Rosen et al., 2002). In this study, the internal consistency was good, with Cronbach’s alpha = 0.95 for the total score, ranging from α = 0.76 to α = 0.93 for the subscales.

#### Female Sexual Function Index

The FSFI (Rosen et al., 2000; Italian adaptation by Filocamo et al., 2014) is a multidimensional self-report measure used to assess female sexual functioning. It comprises 19 items that indicate six domains of sexual functioning: sexual desire, sexual arousal, lubrication, orgasm, satisfaction, and pain. All subscale scores are summed up in a total score. Each item is rated on a scale with a 0–5 or 1–5 range. The Italian adaptation has high internal consistency, with Cronbach’s alpha ranging from α = 0.92 to α = 0.97 for the total sample (Filocamo et al., 2014).

#### Quality of Marital Index

The QMI (Norton, 1983) is a six-item measure of satisfaction in which higher scores indicate higher levels of satisfaction. The items in this measure assess global satisfaction and are rated according to 6- or 10-point Likert scales. The QMI has high internal consistency, with Cronbach’s alpha = 0.96 for men and for women. Although there is no Italian validation of this instrument, it is often used in the literature (e.g., Bonechi & Tani, 2011). In this study, the internal consistency was high, with Cronbach’s alpha = 0.96.

### Statistical Analysis

Most statistical analyses were carried out using the statistical program SPSS version 24, and CFAs were carried out with LISREL 8.80 (Jöreskog & Sörbom, 2006).

To assess the factorial structure of the SIS/SES, we performed the same procedure used for the original validation (Carpenter et al., 2008; Janssen et al., 2002a). The construct validity was assessed by CFAs and by a multigroup CFA. For the CFAs, we analyzed the goodness-of-fit indices of three different models: a simple 10-factor model with 45 items, a “10-in-3” hierarchical model that also contained 45 item scores and 10 subscales, loading on three higher-level factors, and a three-level model. To evaluate the model’s adequacy, we used five fit measures as follows: the χ^2^ to degrees of freedom ratio, non-normed fit index (NNFI), standardized root mean square residual (SRMR), root mean square error of approximation (RMSEA), and comparative fit index (CFI). The χ^2^ test is used to measure fit between sample covariance and fitted covariance matrices (Byrne, 2005), with χ^2^ values with degrees of freedom < 2–3 indicating reasonable fit. To compare the existing model with the independent model, Bentler’s CFI and the Bentler–Bonnett NNFI were used. The two fit measures indicate fit with higher values, with a range of 0 to 1. Generally, a CFI and an NNFI value above 0.95 is preferable (Hu & Bentler, 1999). However, models with CFI and NNFI values above 0.90 also fit well. Because it compensates for model complexity and it is not excessively sensitive to sample size, the Steiger-Lind RMSEA was used. Finally, lower values in SRMR, which is a function of the residuals ranging from 0 to 1, also indicate better fit. Hu and Bentler proposed that values ≤ 0.08 indicate a good fit. Smaller values (with a lower bound of zero) indicate a better fit. In general, an RMSEA of < 0.05 (convention) or < 0.06 (Hu & Bentler, 1999) indicates a good fit. To compare different models, we used Bayes information criterion (BIC), with lower values indicating better fit (Schreiber et al., 2006). In each sample, common rules of thumb have been adopted to define an acceptable sample size, including (a) a minimum sample size of 100 or 200 (Boomsma, 1982, 1985), (b) five or 10 observations per estimated parameter (Bentler & Chou, 1987; Bollen, 1989), and (c) 10 cases per variable (Nunnally, 1978). In particular, in our analysis, the minimum sample size was 602 for older participants in Sample 2 with four to six observations per estimated parameter and with 13 cases per variable.

Gender and age effects were evaluated by conducting a multivariate analysis of variance (MANOVA) between scores in dependent variables (i.e., the SES, SIS1, and SIS2 factors) and scores in independent variables (gender in Sample 1, gender and age in Sample 2). Bonferroni’s correction for multiple testing (*p* = .05/3 = .017; *p* = .05/10 = .005) was used to interpret the results. Effect sizes (Cohen’s *d)* were also respectively reported as 0.20 (small effect), 0.50 (average effect), and 0.80 (large effect; Cohen, 1992).

Although internal consistency was determined by calculating Cronbach’s *α* value (0–1 range, with values > 0.70 indicating good internal reliability) for each of the three factors, the correlations between the three factors (SES, SIS1, and SIS2) were evaluated with Pearson’s correlation.

Correlations between the SIS/SES and other measures were evaluated with Pearson’s correlation coefficient. Bonferroni’s correction for multiple testing (*p* = .05/3 = .017; *p* = .05/10 = .005) was used to interpret the results. Hierarchical regression analysis was used to evaluate the impact of gender, age, and their interaction (inserted at the first step), the SIS/SES scales (inserted at the second step), and two- and three-way interactions between the SIS/SES scales and age or gender (inserted at the last step) on BISF factors and dimensions. An a priori power analysis carried out with GPower was used to determine the sample size of linear regression and correlation analyses: with α = .05, small effect size (*r* = 0.20), and power = 0.80, the required sample size is 191 observations.

## Results

### Sample Characteristics

Because Italy does not have significant ethnic diversity, ethnicity was not described in the demographic information. Note that the main sample was divided into two parts, as mentioned above. Additional demographics can be found in Table 1. In both samples, men were significantly older than women were (*t*(1966) = 13.02, *p* < .001 for Sample 1 and *t*(1290) = 2.17, *p* = .030 for Sample 2).

The majority of both samples had a sexual partner (76% and 85%, respectively), with no significant difference between men and women ($\chi^{2}$(1) = 2.76, *ns*, and $\chi^{2}$(1) = 3.69, *ns*, respectively). Women reported a stronger tendency toward polygamy ($\chi^{2}$(2) = 53.47, *p* < .001 in Sample 1; $\chi^{2}$(2) = 11.92, *p* = .004 in Sample 2). No different levels of education were found in Sample 2, *z* =  − 1.97, *ns*, because Sample 1 comprised undergraduate students. Marital status did not seem to have any significance, $\chi^{2}$(3) = 3.34, *ns*, in Sample 1 and $\chi^{2}$(3) = 2.31, *ns*, in Sample 2. No difference was found in the proportion of gay men and lesbian in the samples, $\chi^{2}$(3) = 8.33, *ns*, in Sample 1 and $\chi^{2}$(3) = 7.83, *ns*, in Sample 2, while, regarding sexual activity, more women than men reported sexual encounters with other sex-partners, $\chi^{2}$(1) = 5.23, *p* = .02. This was true for Sample 1 but not for Sample 2, $\chi^{2}$(1) = 3.72, *ns*.

### Confirmatory Factor Analysis Results for Italian Samples

The first CFA compared SIS/SES results for students (1077 women, 891 men). These analyses are presented in Table 2.

**Table 2:** Confirmatory factor analysis results by gender (Sample 1: Students) and by age group (Sample 2: General population)

Model	Chi-square	df	Chi-square/df	RMSEA	NNFI	CFI	SRMR	BIC
*Women (N = 1077)*								
10 factors	3325.17	900	3.69	.047	.94	.94	.065	9608.91
10-in-3 factor	3436.39	932	3.69	.050	.93	.93	.078	9943.55
3 factors	4464.15	942	4.74	.059	.90	.90	.074	11,041.13
*Men (N = 891)*								
10 factors	2920.14	900	3.24	.050	.93	.94	.067	9033.25
10-in-3 factor	3162.11	932	3.39	.052	.93	.93	.077	9492.58
3 factors	3950.61	942	4.19	.060	.90	.91	.076	10,349.00
*Age group 18–33 (N = 690)*								
10 factors	3178.62	900	3.53	.061	.89	.90	.076	9061.64
10-in-3 factor	3358.6	932	3.60	.061	.89	.90	.081	9450.80
3 factors	4089.75	942	4.34	.070	.86	.87	.082	10,247.31
*Age group 34–75 (N = 602)*								
10 factors	3320.86	900	3.69	.067	.89	.90	.078	9081.09
10-in-3 factor	3545.17	932	3.80	.068	.88	.89	.086	9510.21
3 factors	4172.25	942	4.43	.076	.86	.86	.088	10,201.29

As seen in Table 2, the goodness-of-fit statistics show the simple 10-factor model as the best describer of Italian men’s and women’s SIS/SES scores, with all fit indexes indicating a reasonable fit. However, it showed only a modest improvement over the “10-in-3” model. As for the simple three-factor model, it showed a decrease in model–data consistency (for both men and women) compared to the other models. Also, all of the models tended to fit men’s SIS/SES data slightly better than they did women’s, which is shown by the BIC indexes. Stepwise tests for factorial invariance over sex were conducted to provide information about gender differences, as seen in Table 3.

**Table 3:** Multigroup results by gender (Sample 1: Students) and by age (Sample 2: General population)

Model	Restriction	Chi-square	df	Chi-square/df	RMSEA	NNFI	CFI	SRMR	BIC
Sample 1: students (N = 1968)									
10 factors	Configuration	5937.57	1800	3.30	.048	.93	.94	.065	19,573.62
	Factor loadings	6092.59	1835	3.32	.049	.93	.94	.067	19,993.79
	Residual variance	6608.27	1880	3.52	.051	.93	.93	.071	20,850.37
	Intercepts	6604.9	1915	3.45	.050	.93	.93	.071	21,112.14
	Configuration	6592.52	1864	3.54	.051	.93	.93	.078	20,713.41
10-in-3 factor	Factor loadings	6780.19	1899	3.57	.051	.93	.93	.080	21,166.23
	Residual variance	7276.32	1944	3.74	.053	.92	.92	.084	22,003.26
	Intercepts	7304.03	1979	3.69	.052	.92	.92	.082	22,296.11
	Configuration	8417.6	1884	4.47	.059	.90	.91	.074	22,690.00
3 factors	Factor loadings	8682.62	1926	4.51	.060	.90	.90	.077	23,273.20
	Residual variance	9212.24	1971	4,67	.061	.90	.90	.080	24,143.72
	Intercepts	9226.92	2013	4.58	.060	.90	.90	.080	24,476.57
Sample 2: general population (N = 1692)									
10 factors	Configuration	6496.46	1800	3.61	.064	.89	.90	.078	19,391.56
	Factor loadings	6642.68	1835	3.62	.064	.89	.90	.083	19,788.52
	Residual variance	6829.45	1880	3.63	.064	.89	.90	.088	20,297.67
	Intercepts	6805.14	1915	3.55	.063	.89	.90	.088	20,524.10
	Configuration	6899.95	1864	3.70	.065	.89	.89	.086	20,253.55
10-in-3 factor	Factor loadings	7033.38	1899	3.70	.065	.89	.89	.090	20,637.71
	Residual variance	7231.72	1944	3.72	.065	.89	.89	.094	21,158.43
	Intercepts	7219.99	1979	3.65	.064	.89	.89	.094	21,397.44
	Configuration	8260.57	1884	4.38	.072	.86	.86	.088	21,757.45
3 factors	Factor loadings	8480.37	1926	4.40	.073	.86	.86	.092	22,278.13
	Residual variance	8684.74	1971	4.41	.073	.86	.86	.097	22,804.88
	Intercepts	8700.2	2013	4.32	.072	.86	.86	.097	23,121.22

The pattern in test results suggests that the SIS/SES items’ residual variances, factor loadings, and intercepts are not influenced by gender; equality constraints do not deteriorate the model’s fit. Therefore, the structure of the individual differences within the male and female groups is implied to be equal. The model–data fit for Italian participants’ SIS/SES scores was good when compared to that for American participants, while demonstrating adequacy for preliminary testing of our remaining hypotheses. The 10-factor model’s modest improvement in fit was not worth the change from the theoretically consistent and parsimonious three-factor model for men (Carpenter et al., 2008; Janssen et al, 2002a). Therefore, we used the three-factor model to examine SIS/SES scores from Italian participants.

The second CFA was based on the age difference between the participants (see Table 2). Similarly to the previous statement, the goodness-of-fit statistics in Table 2 indicate that the simple 10-factor model described the SIS/SES scores more accurately for both younger and older Italian participants but only showed marginal improvement over the “10-in-3” factor model, even if fit indexes such as RMSEA and CFI were not sufficiently good for some of the group models. The simple three-factor model showed decreased model–data consistency for both the younger and older participants, and all of the models fit the SIS/SES data slightly better with younger participants than with older ones, as shown by the fit indexes. Stepwise tests for factorial invariance over age were conducted to provide information about age differences (see Table 3).

**Fig. 1:**
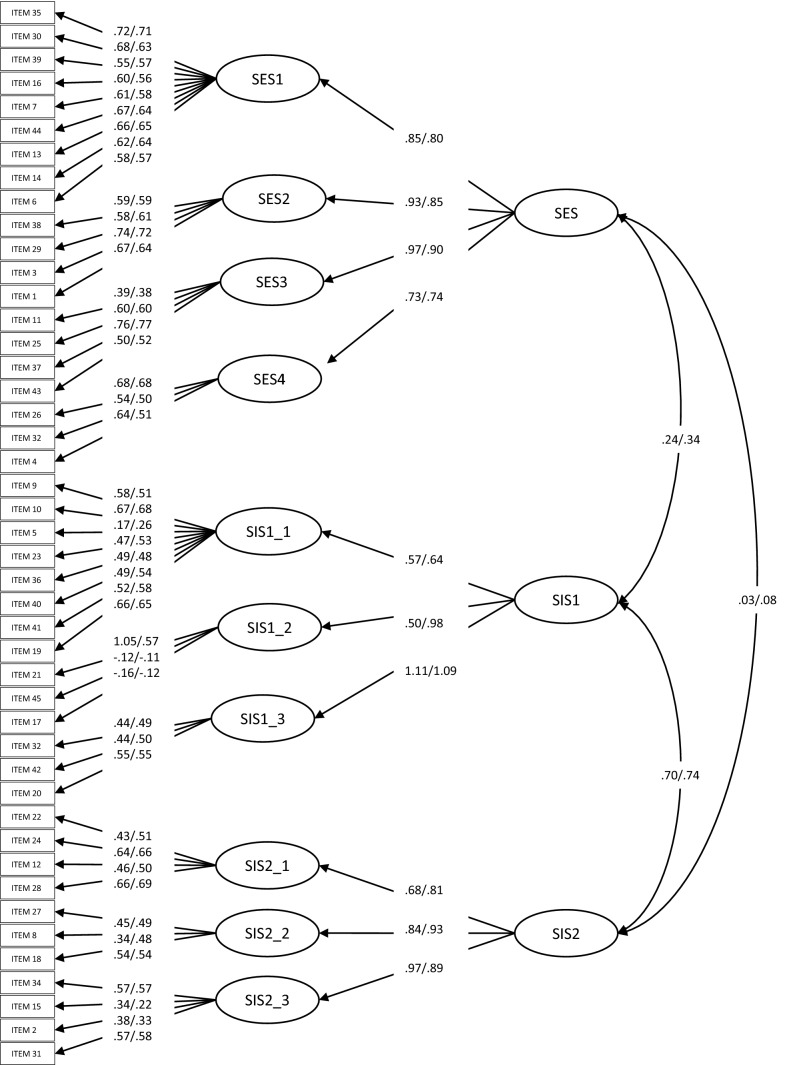
Three factor model of the Italian SIS/SES (sample1/sample2)

SIS/SES items’ residual variances, factor loadings, and intercepts were not influenced by age; moreover, equality constraints did not deteriorate the model’s fit (see Table 3 and Fig. 1). This implies an equal structure of the individual differences within younger and older groups. In both samples and all models, factor loadings were insignificant in two items (45 and 17 on SIS1_2) and one correlation was insignificant (SES and SIS2). The 10-factor model showed only a modest improvement in fit in comparison with the theoretically consistent and parsimonious three-factor model for men (Carpenter et al., 2008; Janssen et al, 2002a, 2002b). Therefore, we used the three-factor model to examine SIS/SES scores from Italian students.

### Gender and Age Comparisons on the SIS/SES Scales

The MANOVAs performed on the three higher-level scales, *F*(3, 1964) = 211,76, *p* < .001, and the 10 lower-level subscales, *F*(10, 1957) = 103.86, *p* < .001, on Sample 1 indicated that the pattern of SIS/SES scale scores was different for male and female participants (see Tables 4 and 5). MANOVA revealed significant gender differences in the higher-level sexual excitation (SES) and inhibition scales (SIS1/Threat of Performance Failure and SIS2/Threat of Consequences). As predicted, men’s SES scores were significantly higher than women’s were, *F*(1, 1966) = 213,67, *p* < .001, while women scored higher than men did on both SIS1, *F*(1, 1966) = 121,34, *p* < .001, and SIS2, *F*(1, 1966) = 333,57, *p* < .001. The effect sizes for gender differences were as follows: *d* = 0.66 for SES, *d* =  − 0.50 for SIS1, and *d* =  − 0.81 for SIS2.

**Table 4:** Gender and age comparisons SES, SIS1, and SIS2 for Sample 1 and Sample 2

	Men (N = 891)	Women (N = 1077)	Gender comparisons (Sample 1)	
	M (SD) *α*	M (SD) *α*	*F*(1, 2006)	d
**SES**: Sexual Excitation (20 items)	50.06 (10.81) .90	42.98 (10.6) .91	213.67^(a)^	.66
**SIS1**: Inhibition due to Threat of Performance Failure (14 items)	27.83 (6.46) .79	30.84 (5.66) .69	121.34^(a)^	− .50
**SIS2**: Inhibition due to Threat of Performance Consequences (11 items)	27.66 (5.24) .67	32.17 (5.89) .71	333.57^(a)^	− .81

	Men (N = 611)	Women (N = 681)	Gender Comparisons (Sample 2)	
	M (SD) *α*	M (SD) *α*	*F*(1, 1288)	d
**SES**: Sexual Excitation (20 items)	48.83 (10.71) .89	45.17 (10.64) .89	36.22^(a)^	.34
**SIS1**: Inhibition due to Threat of Performance Failure (14 items)	27.70 (7.38) .82	32.07 (6.21) .72	135.75^(a)^	− .64
**SIS2**: Inhibition due to Threat of Performance Consequences (11 items)	27.94 (5.91) .71	31.86 (6.23) .75	127.52^(a)^	− .65

	Men Age group 18–33 (N = 305)	Men Age group 34–75 (N = 306)	Age Comparisons (Sample 2)	
	M (SD) *α*	M (SD) *α*	*F*(1, 609)	d
**SES**: Sexual Excitation (20 items)	49.15 (10.13) .88	48.52 (11.26) .90	.53	.06
**SIS1:** Inhibition due to Threat of Performance Failure (14 items)	27.63 (7.21) .81	27.77 (7.54) .83	.06	− .02
**SIS2:** Inhibition due to Threat of Performance Consequences (11 items)	28.09 (5.90) .72	27.88 (5.93) .72	.34	.05

	Female Age group 18–33 (N = 385)	Female Age group 34–75 (N = 296)	Age Comparisons(Sample 2)	
	M (SD) *α*	M (SD) *α*	*F*(1, 679)	d
**SES**: Sexual Excitation (20 items)	44.75 (10.43) .89	45.72 (10.90) .89	1.38	− .09
**SIS1:** Inhibition due to Threat of Performance Failure (14 items)	31.75 (5.96) .72	32.50 (6.51) .71	2.45	− .12
**SIS2:** Inhibition due to Threat of Performance Consequences (11 items)	32.39 (5.45) .39	31.18 (7.07) .80	6.36^(b)^	.20

**Table 5:** Gender and age differences on lower-level SIS/SES subscales (Sample 1 and Sample 2)

	Men (N = 891) M (SD)	Women (N = 1077) M (SD)	Gender comparisons (Sample 1) *F*(1, 1968)	*d*
**SES**: “Social interactions” (9 items)	23.12 (5.64)	19.13 (5.59)	245.53^**(a)**^	.71
“Visual stimuli” (4 items)	9.72 (2.58)	7.63 (2.72)	302.09^**(a)**^	.79
“Fantasizing about sex” (4 items)	11.54 (2.42)	11.40 (2.42)	1.56	.06
“Nonspecific Stimuli” (3 items)	5.68 (2.24)	4.81 (1.88)	88.33^**(a)**^	.42
**SIS1**: “Losing arousal easily” (8 items)	16.32 (4.61)	18.13 (4.11)	79.19^**(a)**^	− .41
“Partner concerns” (3 items)	4.90 (1.43)	5.54 (1.57)	92.21^**(a)**^	− .43
“Performance concerns” (3 items)	6.55 (2.09)	7.17 (1.97)	45.67^**(a)**^	− .31
**SIS2**: “Risk of being caught” (4 items)	10.02 (2.66)	12.05 (2.70)	280.09^**(a)**^	− .76
“Negative consequence” (3 items)	7.61 (2.01)	8.77 (2.19)	146.81^**(a)**^	− .55
“Pain/norms and values” (4 items)	10.03 (2.49)	11.35 (2.55)	133.37^**(a)**^	− .52

	Men (N = 611) M (SD)	Women (N = 681) M (SD)	Gender comparisons (Sample 2) *F*(1, 1288)	*d*
**SES**: “Social interactions” (9 items)	21.86 (5.67)	19.00 (5.74)	81.44^**(a)**^	.50
“Visual stimuli” (4 items)	9.99 (2.71)	8.96 (2.99)	38.85^**(a)**^	.36
“Fantasizing about sex” (4 items)	11.36 (2.72)	11.79 (2.48)	9.23^**(b)**^	− .17
“Nonspecific Stimuli” (3 items)	5.61 (2.12)	5.43 (2.07)	1.98	.09
**SIS1:** “Losing arousal easily” (8 items)	16.35 (5.00)	19.02 (4.56)	102.18^**(a)**^	− .56
“Partner concerns” (3 items)	4.97 (1.44)	5.72 (1.64)	78.99^**(a)**^	− .49
“Performance concerns” (3 items)	6.38 (2.27)	7.33 (2.05)	62.66^**(a)**^	− .44
**SIS2:** “Risk of being caught” (4 items)	10.06 (2.99)	11.71 (2.99)	94.99^**(a)**^	− .55
“Negative consequence” (3 items)	7.52 (2.23)	8.62 (2.45)	65.77^**(a)**^	− .47
“Pain/norms and values” (4 items)	10.36 (2.54	11.52 (2.42)	68.05^**(a)**^	− .47

	Men—Age group 18–33 (N = 305) M (SD)	Men—Age Group 34–75 (N = 306) M (SD)	Age comparisons (Sample 2) *F*(1, 609)	*d*
**SES**: “Social interactions” (9 items)	22.37 (5.50)	21.35 (5.73)	4.98^(b)^	.18
“Visual stimuli” (4 items)	9.82 (2.45)	10.16 (2.94)	2.47	− .13
“Fantasizing about sex” (4 items)	11.45 (2.56)	11.28 (2.88)	.64	.06
“Nonspecific Stimuli” (3 items)	5.50 (2.12)	5.72 (2.12)	1.65	− .10
**SIS1**: “Losing arousal easily” (8 items)	16.35 (4.91)	16.36 (5.09)	.00	.00
“Partner concerns” (3 items)	4.93 (1.38)	5.02 (1.51)	.59	− .06
“Performance concerns” (3 items)	6.35 (2.28)	6.40 (2.25)	.05	− .02
**SIS2**: “Risk of being caught” (4 items)	10.12 (2.96)	10.01 (3.02)	.23	.04
“Negative consequence” (3 items)	7.59 (2.19)	7.45 (2.26)	.58	.06
“Pain/norms and values” (4 items)	10.38 (2.53)	10.35 (2.55)	.02	.01

	Female–Age group 18–33 (N = 385) M (SD)	Female–Age group 34–75 (N = 296) M (SD)	Age comparisons (Sample 2) *F*(1, 679)	*d*
**SES**: “Social interactions” (9 items)	19.07 (5.75)	18.90 (5.73)	.14	.03
“Visual stimuli” (4 items)	8.69 (2.93)	9.30 (3.04)	7.12^(b)^	− .21
“Fantasizing about sex” (4 items)	11.69 (2.35)	11.93 (2.65)	1.52	− .10
“Nonspecific Stimuli” (3 items)	5.31 (2.01)	5.59 (2.15)	3.10	− .14
**SIS1**: “Losing arousal easily” (8 items)	18.84 (4.21)	19.26 (4.97)	1.46	− .09
“Partner concerns” (3 items)	5.60 (1.58)	5.88 (1.69)	4.83^(b)^	− .17
“Performance concerns” (3 items)	7.31 (2.04)	7.35 (2.06)	.09	− .02
**SIS2**: “Risk of being caught” (4 items)	11.82 (2.73)	11.57 (3.30)	1.09	.08
“Negative consequence” (3 items)	8.90 (2.34)	2.27 (2.55)	11.27^(a)^	.26
“Pain/norms and values” (4 items)	11.68 (2.23)	11.34 (2.64)	3.26	.14

**(a)** *p* < .001; **(b)** *p* < .050 (SES = Sexual Excitation, SIS1 = Sexual Inhibition-1, SIS2 = Sexual Inhibition-2); Sample 1: Students; Sample 2: General population

MANOVAs performed on the three higher-level scales—*F*(3, 1286) = 97.51, *p* < .001 for gender; *F*(3, 1286) = 3.38, *p* = .018 for age; and *F*(3, 1286) = 1.69, *p* = .167 for interaction—and the 10 lower-level subscales—*F*(10, 1279) = 50.60, *p* < .001 for gender; *F*(10, 1279) = 4.75, *p* < .001 for age; and *F*(10, 1279) = 1.17, *p* = .306 for interaction—on Sample 2 indicated that the pattern of SIS/SES scales scores differed for male and female participants and that only SIS2 scores differed for younger and older participants (see Tables 4 and 5). No interaction existed between age and gender. MANOVAs revealed significant gender differences on the higher-level sexual excitation (SES) and inhibition scales (SIS1/Threat of Performance Failure and SIS2/Threat of Consequences). As predicted, men’s SES scores were significantly higher than women’s were, *F*(1, 1966) = 213,67, *p* < .001, while women scored higher than men did on both SIS1, *F*(1, 1966) = 121,34, *p* < .001, and SIS2, *F*(1, 1966) = 333,57, *p* < .001. The effect sizes for gender differences were as follows: *d* = 0.34 for SES, *d* =  − 0.64 for SIS1, and *d* =  − 0.65 for SIS2. A MANOVA revealed significant age differences in the higher-level SIS2/Threat of Consequences. Only younger female participants’ SIS1 scores were significantly higher than those of older female participants were *F*(1, 609) = 6.36, *p* = .012, while there was no difference in SES, *F*(1, 1288) = 0.08, *ns*, or SIS1, *F*(1, 1288) = 1.39, *ns*. The effect size for age differences in SIS2 was *d* = 0.15.

### Relationships Between the SIS/SES Scales 

Men’s scores (*n* = 891 for Sample 1, *n* = 611 for Sample 2) on the three higher-level scales (SES, SIS1, and SIS2) showed a significant correlation between SES and SIS1, *r* = 0.31, *p* < .001 for Sample 1; *r* = 0.40, *p* < .001 for Sample 2, while the relationship between SES and SIS2 for men was negligible, *r* = 0.01, *ns*, for Sample 1 and *r* = 0.10, *ns*, for Sample 2. Similar results were found for women (*n* = 1,077 for Sample 1, *n* = 681 for Sample 2), whose scores on the three higher-level scales (SES, SIS1, and SIS2) showed a significant correlation between SES and SIS1 (*r* = 0.24, *p* < .001 for Sample 1; *r* = 0.20, *p* < .001 for Sample 2), while the relationship between SES and SIS2 was negligible, *r* =  − 0.02, *ns*, for Sample 1 and *r* = 0.06, *ns*, for Sample 2. The SIS1 and SIS2 scales were significantly correlated, both among men, *r* = 0.35, *p* < .001 for Sample 1; *r* = 0.41, *p* < .011 for Sample 2, and in women, *r* = 0.37, *p* < .001 for Sample 1; *r* = 0.24, *p* < .011 for Sample 2. This correlation was slightly more pronounced in men in Sample 2 and reflects a significant gender difference. According to these statistics, the three higher-level SIS/SES scales were relatively independent for both women and men.

Older participants’ scores (*n* = 602) on the three higher-level scales (SES, SIS1, and SIS2) showed a significant correlation between SES and SIS1 for participants aged 34–75 years, *r* = 0.31, *p* < .001, and were similar to younger participants’ (*n* = 690) scores, *r* = 0.25, *p* < .001. The correlation between SES and SIS2 for both the older and younger groups was negligible, *r* =  − 0.09, *ns*, and *r* =  − 0.05, *ns*, respectively. The correlation was slightly more pronounced for older participants and reflects a significant age difference. According to these statistics, the three higher-level SIS/SES scales were relatively independent for both older and younger participants.

### Relationship of Factor Scores to Age

In Samples 1 and 2, the SES and SIS1 scales showed a significant positive correlation with age for both men and women (see Table 6). Only in older women (Sample 2) did SIS2 show a positive correlation with age, *r* = 0.21, *p* < .001. Multiple regression analysis showed no SIS or SES factor to be related to age. There was a significant age × gender interaction on SIS2 with a negative effect of the age in women, $\beta$  =  − 0.25, *p* < .01, *r*^2^ = 0.11, *p* < .001.

**Table 6:** SES-SIS correlation by age in Sample 2 (Age 34–75 in upper triangle)

Gender	Variable	SES	SIS1	SIS2
Male	SES	–	.42*	.04
	SIS1	38*	–	.28*
	SIS2	.17	.54*	–
Female	SES	–	.31*	.21*
	SIS1	.30*	–	.46*
	SIS2	− .08	.38*	−
Total	SES	–	.31*	−.09
	SIS1	.25*	−	.41*
	SIS2	− .05	.52*	–

*p < *.*001

### Convergent and Discriminant Validity

The correlations between SES, SIS1, SIS2, and other measures for women and men in Sample 2 were calculated for 1,222 participants who completed the BISF (16 were excluded for incomplete or incoherent answers) and 195 participants who completed the QMI. For the BISF, we explored the relationship between three scales of the SIS/SES and age, as independent variables, and four factors and seven dimensions of the BISF. Table 7 shows the average scores and the factors that contribute to explaining sexual functions in both men and women. For the QMI, no correlation was found between this scale and SIS2 for either men or women, while negative correlations were found between this scale and both SES and SIS1 in men, *r* =  − 0.34, *p* < .01 and r =  − 0.22, *p* < .05, respectively, but not in women, *r* = 0.01, *ns*, and *r* =  − 0.06, *ns*, respectively.

**Table 7:** Average scores and predictors of BISF, IIEF, and FSFI

Dependent variable	Male	Female	Predictors
*BISF*			
F1- Couple Sexuality	3.06	2.93	age^−^,SES^+^, SIS1^−^
F2- Autoeroticism	2.99	2.01	gen^−^, age^−^, SES^+^
F3- Unsatisfaction	1.04	1.22	SIS1^+^
F4- Anal Sexuality	1.86	0.74	gen^−^, age^−^, SES^+^, SIS1^−^, SIS2ˉ
D1- Thoughts/desires	7.27	5.31	gen^−^, age^−^, SES^+^, SIS1^−^, SIS2ˉ
D2- Arousal	6.96	6.22	SES^+^, SIS1^−^
D3- Frequency	4.50	4.30	age^−^, SES^+^, SIS1^−^
D4- Receptivity/initiation	9.49	7.95	gen^−^, SES^+^, SIS1^−^
D5- Pleasure	4.47	3.80	SES^+^, SIS1^−^
D6- Satisfaction	8.22	8.20	SES^+^, SIS1^−^
D7- Problems	3.01	3.69	gen^+^, SIS1^+^
*IIEF*			
Erectile Function	23.05		
Orgasmic Function	7.67		
Sexual Desire	7.95		
Intercourse satisfaction	9.36		
Overall satisfaction	7.21		SIS1^−^
Total score	55.95		
*FSFI*			
Sexual desire		6.96	SES^+^
Sexual arousal		14.74	
Lubrication		16.26	
Orgasm		10.64	
Satisfaction		10.73	
Pain		10.99	
Total score		70.50	

Results of multiple regression analysis with gender (female), age, SES, SIS1, SIS2, as independent variables for BISF, IIEF and FSFI. Average scores for male and female are reported, and significant predictors (*p* < .01) with + and – signs represent the direction of the relationship

### SIS*/*SES and Sexual Functioning

In total, 118 men completed the IIEF (four were excluded for incomplete answers) and 121 women completed the FSFI (four were excluded for incomplete answers). We explored the relationship between the three scales of the SIS/SES and age, as independent variables, and both five factors of the IIEF and total score for the male sample and six factors of the FSFI and total score for the female sample. Table 7 shows average scores and the factors that contribute to explain sexual functions.

## Discussion

The present study had the objective of validating the SIS/SES on an Italian sample, to evaluate its validity and effectiveness concerning sexual functioning in men and women in the country. The statistical analysis showed a positive fit for the three-factor model, highlighting differences found in previous studies (Carpenter et al., 2011; Janssen & Bancroft, 2007; Janssen et al., 2008), according to which women tended to have higher indexes in factors related to sexual inhibition, while men did so for factors related to sexual excitation. Even in the Italian sample, despite possible cultural differences, SIS/SES proved to be valid tools for measuring aspects related to human sexuality.

Concerning the factorial analysis, we expected to find no significant difference between the American and Italian samples in the factorial structure of the SIS/SES. Our factorial analysis results matched the results obtained from the original validation sample (Carpenter et al., 2008, 2011; Janssen et al., 2002a). Based on the fit indices alone, we concluded that a 10-factor model could also be better for an Italian sample, especially for aspects related to age differences, while offering a marginal improvement to fit indices for both male and female samples. A second analysis was used to test the “10-in-3” model, which, while showing a slight improvement in the model’s fit indexes, did not justify sacrificing the practicality and simplicity of the three-factor model proposed by the Janssen et al. (2002a), since the latter showed adequate and sufficiently good indexes to discriminate differences related to the excitation/inhibition model in both men and women. Contrasting with the Finnish (Varjonen et al., 2007) and the Spanish versions (Granados et al., 2018) but in line with the Portuguese version (Quinta Gomes et al., 2018), the Italian version of the SIS/SES encompasses all of the scales’ original items (Carpenter et al., 2008; Janssen et al., 2002a), confirming the measurement validity in assessing sexual excitation and inhibition systems, in both clinical and research settings. Moreover, the CFA found in Table 2 shows that the three-factor model showed good indexes for both men and women, with modest decreases in fit for the three-factor model and the “10-in-3” factor models, but that were not relevant enough to justify abandoning the model Janssen et al. (2002a) selected. The test factorial invariance (Table 3) suggested that the structure of individual differences in SIS/SES scores was the same for men and women. The results confirmed both the models’ configural invariance across men and women and the equality of the factor loadings, residual variances, and thresholds across groups. Indeed, no intraindividual differences related to gender were found. Analysis of variance for the three-factor model suggested that the complex of individual differences between men and women remained unchanged. Only specific men with greater significant activation of the sexually dimorphic area and amygdala (the area located in the hypothalamus known to play a pivotal role in physiological arousal and sexual behavior) had strongly elevated sexual arousal and excitation with respect to women (Chivers et al., 2004; Hamann et al., 2004; Karama et al., 2002). Therefore, we could claim the existence of interindividual variability, which should be studied in a clinical and therapeutic context.

Men, regardless of age, showed higher scores than women in sexual excitation, while women showed significantly lower scores, although the effect size was lower for younger participants. The differences continued for aspects related to inhibition: as predicted, men showed significantly lower scores in both the SIS1 and SIS2 factors than women did. These results were in line with the hypothesis and seemed to be a long-term female trait (Velten et al., 2017). These differences could be explained by biological and evolutionary aspects, in that environmental conditions forced women to inhibit their excitation, to avoid wasting reproductive potential with men that do not show adequate resources (Buss, 2007; Gangestad, 2006). According to the evolutionary perspective, for men, on the other hand, motivations are a direct consequence of this adaptive relationship between environment and reproductive drive. Because men, from an evolutionary perspective, do not have to participate in gestation and caregiving, they do not need to inhibit their fast and immediate excitation, instead favoring mating and enhancing reproductive possibilities (Buss & Strategies, 2002; Fontanesi & Renaud, 2014; Shackelford & Goetz, 2007; Symons, 1979). Moreover, many have had emphasized the differences between men and women at the level of arousal and excitation (Hamann et al., 2004). From a neurophysiological point of view, evidence suggests clear sexual dimorphism in many areas of the brain (Rupp & Wallen, 2008). Despite this dimorphism, we observed that the anterior cingulate, medial prefrontal, orbitofrontal, insular, and occipitotemporal cortices as well as the amygdala and ventral striatum were involved in sexual arousal and excitation in both men and women (Chivers et al., 2004; Rupp & Wallen, 2008).

Interestingly, the Italian sample scored lower than the validation sample did in the SES scale but obtained similar results to those of the validation sample in the SIS scales. While is impossible to compare this data with those of other Mediterranean population validations (such as for the Spanish population) due to the differences in the number of items, we can suggest that the propensity for Italian participants to rate SES items lower is due to cultural effects, shaping attitudes, behavior, and personality. Micò et al. (2013) suggested that inhibitory aspects have deeper effects on sexual motivation than excitation ones do, and the same results have been obtained when analyzing psychosocial variables affecting sexual drive (Nimbi et al., 2019). Sexual conservatism, sexism, and social anxiety in Italian male samples and religion, adherence to sexual roles, fear of negative evaluation, and poor sexual education in Italian women strongly influence sexual arousal and attitudes (Nimbi et al., 2019; Panzeri & Fontanesi, 2013). Nevertheless, cross-cultural investigations could be helpful to better understand the differences in SES scores between Italian and other countries’ samples.

Concerning age differences, we expected to find a globally similar factor structure underlying sexual processes in both older and younger participants. Several studies have shown that despite differences in hormone production, regarding sexual arousal in men and women, the neurobiological circuits that regulate sexual behavior do not change with age (Kafka, 1997; Levine, 2003; Pfaus & Everitt, 1995).

The CFA found in Table 2 indicated that the three-factor model showed adequate indexes for both younger and older participants, with modest decreases in fit for the three- and “10-in-3” factor models, but was not relevant enough to justify the abandonment of our selected model. The test factorial invariance (Table 3) suggested that the structure of individual differences in SIS/SES scores was the same for younger and older participants. The results confirmed both the configural invariance of models across younger and older participants and the equality of factor loadings, residual variances, and thresholds across groups.

In the current study on older and young adults, the Italian sample did not show significant age-related differences in SIS1 and SES scores, unlike in previous studies, such as those by Janssen et al. (2002a) and by Pinxten and Lievens (2014). Also unlike previous studies (Carpenter et al., 2008; Janssen et al., 2002a), we found a significant positive correlation between SES and SIS1 (regarding inhibition due to the threat of performance failure). Future studies should take this unusual result into account, but this relationship could be related to the meaning of the items included in the SIS1. The SIS1 items focus on the role of thoughts, sounds, and external factors that can negatively influence sexual arousal and sexual performance, while SES focuses on the relevance of sexual thoughts during sexual activities. We can suggest that the more a person fears the threat of performance failure, due to external causes or nonsexual thoughts, the more they need to stay focused on sexual fantasies and sensations to maintain their arousal. The literature has found negative influences of nonsexual thoughts during any sex-related activity on sexual pleasure and orgasm for both men (Purdon & Watson, 2011) and women (Cuntim & Nobre, 2011). Moreover, a similar result was found in the younger sample (mean age = 20.38) studied by Granados et al. (2018). The Spanish socio-cultural environment is close to the Italian one, and female sexuality has recently faced a progressive masculinization, especially in young girls. To support this, we only found age differences regarding inhibition due to the threat of negative consequences (the second factor of SIS2) among women. These differences perhaps indicate that young women feel emotionally sensitive to the consequences of failure, particularly to three specific themes: the negative effects of sexuality on reputation, the fear of pain, and the risk of being caught. A growing idea shared by female students during classroom sex education is that female satisfaction, as men’s is, lies only in the achievement of orgasm and not in the psychological and physical pleasure of sharing an intimate moment with a partner. Failing to orgasm can be perceived as a sign of “sexual malfunction,” even in women. Besides, especially in the Italian context, the conviction that a “real orgasm” is connected to female ejaculation is spreading among young girls due to the trending book *Female Ejaculation and the G-Spot* by Sundahl (2003). This orgasm-oriented, mechanical vision of sexuality can be the reason behind the unexpected relationship between SIS1 and SES and help to explain how young girls perceive such negative pressure regarding sexual activities.

Contrarily to our hypothesis, aspects related to sexual function found through the FSFI and IIEF questionnaires showed a very poor significant correlation with factors investigated by the SIS/SES. We cannot yet explain these results. Maybe FSFI and IIEF are generic measures of the frequency of possible sexual problems that, without perceived clinical suffering, are not to be considered real sexual dysfunctions. A study on patients with clinical diagnoses is needed to clarify this point. Other sociocultural aspects typical of Italian society may be involved in these results and should be taken into account in future studies. Only the questionnaire on marriage quality showed negative correlations with the male sample but not with the female sample. This could mean that in a long-term relationship, as confirmed by previously cited evolutionary hypotheses, sex is a fundamental aspect for men and a marginal aspect for women. However, Table 7 shows factors—SIS1, SIS2, SES, age, and gender—that could contribute to explaining different aspects of sexuality revealed by the BISF. The excitation factor influenced couple eroticism, autoeroticism, and anal sexuality, without specific differences between men and women. As claimed in the hypothesis, it also influenced all of the positive aspects related to sexuality, specifically desires, excitation, frequency, receptivity, pleasure, and satisfaction. As expected, female gender, age, and inhibition influenced dissatisfaction, suggesting that aspects related to dissatisfaction were strongly dependent on the couple relationship. In the same way, anal sex—which was still seen as taboo—was influenced by gender (it was more related to male sexuality) and, as expected, by SIS1 and SIS2. Finally, regarding problematic characteristics of sexual function, female gender and older age influenced this aspect, along with the inhibition found by SIS1. These data support the use of SIS/SES as a reliable instrument to assess different aspects of human sexuality, especially those concerning social, psychological, and biological aspects.

In addition to what has already been described, our results have relevant implications in the clinical setting. The validation of the SIS/SES instrument highlights the interindividual differences in sexual arousal and motivation in the target population. Specifically, the results show differences in excitation between men and women, regardless of age but connected to the length of the relationship. Among long-term married couples, men still have fast and immediate excitation, while sexual arousal decreases in women. This aspect has important clinical implications and must be addressed during sexual couple therapies, to both normalize and contextualize this discrepancy within the therapeutic setting. Moreover, we found evidence that both men and women, especially young participants, are living their sexual lives focusing on sexual performance, rather than on relationships, sensations, and emotions. This aspect can have negative implications on the development of adequate and satisfactory sexuality in adult life and also on the onset of sexual disorders. Furthermore, sexual disorders are strictly connected to the sexual-inhibition and sexual-excitation systems, as reported by the cited literature, and the SIS/SES has proven to be relevant measures in the clinical setting with which to record specific aspects connected to sexual disorders’ symptomatology.

### Limitations and Future Studies

Our research suffered from several limitations. First, all of the collected data were self-reported and may be subject to biases, especially concerning sexuality. Secondly, longitudinal studies are recommended to study age effects and to disentangle cohort effects from age effects. Moreover, our results also should be validated among in-patient samples with a sexual-related diagnosis. Finally, future research should address a larger number of homosexual participants to investigate sexual-orientation differences regarding SIS/SES. Despite these limitations, we believe that our results could be useful in both clinical and scientific contexts.

### Conclusion

This work aimed to validate the SIS/SES questionnaires and their related theoretical model in two samples of men and women in Italy. In terms of statistical validity, the three-factor questionnaire is a great tool for measuring sexuality in Italy. Indexes testing this model’s goodness of fit have highlighted not only its effective validity but also its effectiveness in evaluating differences between men and women as well as individual variability, as other international studies have found (Carpenter et al., 2008; Pinxten & Lievens, 2014; Quinta Gomes et al., 2018; Velten et al., 2018). This analysis shows adequate and sufficiently good indexes to discriminate differences related to the excitation/inhibition model for both men and women.

The factor structure was similar for men and women, regardless of age, although men scored significantly higher on sexual excitation. Moreover, we observed interindividual variability, which is very useful for clinical and therapeutic contexts.

In line with existing literature in other countries (Carpenter et al., 2008; Granados et al., 2018; Pinxten & Lievens, 2014; Quinta Gomes et al., 2018; Varjonen et al., 2007; Velten et al., 2018), our data underlie this model’s relevance to studying sexual behavior, filling an existing gap in sexological research and therapy in Italy. In conclusion, through analysis of the results for the SIS/SES questionnaire, the present work was aimed at contributing to the scientific thread viewing the dual-control model as one of the main theories in the study of sexuality, by confirming its validity and effectiveness.
